# Rapid Profiling of EEG Responses to Non-Invasive Brain Stimulation in Parkinson’s Disease: A Biomarker-Driven Screening Framework

**DOI:** 10.3390/biomedicines14020352

**Published:** 2026-02-03

**Authors:** Sepideh Hajipour Sardouie, Mahdi Babaei, Mahsa Naseri, Shervin Mehrtash, Mohamad Hosein Faramarzi, Zahra Kavian, Martin S. Keung, Varsha Sreenivasan, Hanaa Diab, Maryam S. Mirian, Martin J. McKeown

**Affiliations:** 1 Pacific Parkinson’s Research Centre, Djavad Mowafaghian Centre for Brain Health, University of British Columbia, Vancouver, BC V6T 2B5, Canada; sepideh.hajipour@ubc.ca (S.H.S.); mahdi78babaei@gmail.com (M.B.); naserimahsa3@gmail.com (M.N.); ishervinmehrtash@gmail.com (S.M.); faramarzimohamadhosein@gmail.com (M.H.F.); zahra.kavian@ubc.ca (Z.K.); martinksm@hotmail.com (M.S.K.); varsha.sreenivasan@ubc.ca (V.S.); hanaaadiab@gmail.com (H.D.); maryam.mirian@ubc.ca (M.S.M.); 2 Faculty of Medicine (Neurology), University of British Columbia, Vancouver, BC V6T 2B5, Canada

**Keywords:** Parkinson’s disease (PD), EEG biomarkers, resting-state, galvanic vestibular stimulation (GVS), neuromodulation, multi-criteria decision analysis (MCDA)

## Abstract

**Background/Objectives**: Parkinson’s disease (PD) is associated with alterations in resting-state Electroencephalogram (EEG) biomarkers. Identifying stimulation protocols that reliably shift these biomarkers toward healthy-like patterns is essential for developing personalized neuromodulation strategies. This study introduces a rapid, biomarker-driven framework for screening the EEG effects of diverse Galvanic Vestibular Stimulation (GVS) waveforms in PD. **Methods**: More than 300 subthreshold GVS stimuli were delivered during resting-state EEG to PD (*n* = 5) subjects and Healthy Controls (*n* = 5). A composite biomarker score that included spectral, cross-frequency, aperiodic, and complexity measures quantified stimulation-related changes. A linear classifier and multi-criteria decision analysis were used to evaluate and rank stimuli. **Results**: Stimulation produced consistent improvements in the composite biomarker score, with the strongest effects observed for beta-range sinusoids, multisine waveforms, frequency-modulated stimuli with a 75 Hz carrier, and PAC-modulated signals. No significant post-stimulation carryover effects were detected. **Conclusions**: While preliminary, this exploratory framework enables rapid, interpretable profiling of EEG responses to non-invasive stimulation in PD. By prioritizing candidate GVS protocols based on biomarker shifts rather than behavioural endpoints, the approach provides a practical foundation for future personalized neuromodulation strategies.

## 1. Introduction

Parkinson’s disease (PD) is a progressive neurodegenerative disorder whose cardinal motor features, i.e., bradykinesia, rigidity, tremor, and postural instability, reflect dysfunction across basal ganglia–thalamocortical circuits and large-scale cortical networks. While dopaminergic medications and invasive approaches such as deep brain stimulation (DBS) provide important symptomatic relief for many patients, these therapies are limited by variable efficacy, side effects, surgery-related risks, and a persistent need for individualized dosing and parameter selection. Recent reviews on PD therapeutics have emphasized the importance of more personalized, less invasive interventions [1].

Resting-state electroencephalogram (EEG) provides a noninvasive, temporally precise window onto the oscillatory and network alterations associated with PD. Converging evidence indicates consistent abnormalities in spectral power (notably in the beta band), altered cross-frequency interactions such as beta–gamma phase–amplitude coupling (PAC), reduced signal complexity, and disrupted functional connectivity. These features relate to both motor and cognitive symptoms and can serve as objective biomarkers of disease state and progression. Because these biomarkers can be acquired rapidly at rest and are sensitive to moment-to-moment neural state, they are well-suited as scalable markers for screening and optimizing neuromodulatory interventions [2,3,4].

Building on this potential for biomarker-guided neuromodulation, recent efforts have explored complementary stimulation techniques that can directly modulate sensorimotor circuits. Galvanic vestibular stimulation (GVS), i.e., the application of low-intensity currents to the mastoid region to engage vestibular afferents, has emerged as a flexible, noninvasive neuromodulation modality with potential utility in motor disorders. A growing body of clinical and mechanistic work shows that GVS can influence postural control, gait stability, and sensorimotor integration. These effects are strongly dependent on stimulation parameters (waveform, frequency content, amplitude, duration) and electrode configuration. Noisy (stochastic) GVS in particular has attracted attention because it may exploit stochastic resonance to enhance weak vestibular signals, although results are mixed and highlight the need for careful parameter optimization and individualized dosing. Recent comprehensive reviews and controlled studies emphasize both the promise of GVS and the importance of rigorous, parameter-specific evaluation [5,6,7,8].

Systematically testing behavioural outcomes for hundreds of candidate stimulation waveforms in clinical populations is impractical: it is time-consuming, fatiguing for participants, and costly. Moreover, behavioural responses are inherently noisy and variable, requiring longer trial durations and multiple repetitions per stimulus to reliably detect effects. This dramatically increases the burden on both participants and researchers, especially when testing large waveform libraries of potential stimuli. To accelerate identification of candidate protocols while preserving biological relevance, we propose a rapid, EEG-driven screening pipeline. The central assumption of this approach is tractable and testable: GVS waveforms that shift PD patients’ resting-state EEG biomarkers toward the distribution observed in healthy controls mark stimuli that are promising for subsequent behavioural validation. By scoring stimuli according to their capacity to move composite EEG biomarker profiles toward that of healthy controls, one can prioritize a much smaller set of waveforms for rigorous motor outcome testing, substantially reducing the search space.

To demonstrate feasibility, we conducted a high-throughput screening study in which over 300 distinct subthreshold GVS waveforms (sinusoidal, amplitude-modulated, frequency-modulated, multisine [9,10], and PAC-modulated waveforms) were delivered while resting EEG was recorded in PD participants and healthy controls. A diverse set of previously established, PD-relevant EEG biomarkers was extracted, spanning oscillatory features (beta-band power and beta–gamma phase–amplitude coupling [3,11]), beta burst characteristics [12,13], aperiodic spectral components [14,15], signal-complexity measures (e.g., entropy-based indices) [16,17], and beta waveform-shape metrics [18,19]. These biomarkers have been widely linked to abnormal synchronization, altered excitation-inhibition balance, and reduced neural adaptability in PD, providing a multidimensional representation of cortical dynamics. A sparse linear model (LASSO) was trained to discriminate PD from control resting EEG, yielding a composite score that quantified stimulation-related changes in this multivariate biomarker space. Each stimulus trial was then evaluated by its capacity to move the composite biomarker vector toward healthy reference values; clustering and nonparametric statistics were then used to identify stimulus classes that produced consistent, favorable biomarker shifts across participants despite sparse per-stimulus sampling.

Our contributions are: (1) an EEG-based, high-throughput pipeline for prioritizing noninvasive stimulation protocols in PD; (2) identification of stimulus classes that consistently shift EEG biomarkers toward healthy norms; (3) transparent reporting of biomarker contributions to support interpretability of the screening framework; and (4) a practical path to reduce the stimulus space for downstream motor validation and future individualized therapeutic selection. By integrating well-validated EEG biomarkers, principled stimulation parameterization, and machine learning models, this framework aims to speed the translation of noninvasive neuromodulation into more personalized therapeutic strategies for Parkinson’s disease.

This paper is organized as follows. Section 2 describes the experimental design, participant groups, stimulation protocols, EEG acquisition procedures, the biomarker extraction pipeline, the construction of the composite score, and the analytical framework used to evaluate stimulation effects. Section 3 presents the main results, including biomarker changes and stimulus ranking outcomes. Section 4 discusses the implications and limitations of this exploratory study, and Section 5 concludes with directions for future work.

## 2. Materials and Methods

**Figure 1:**
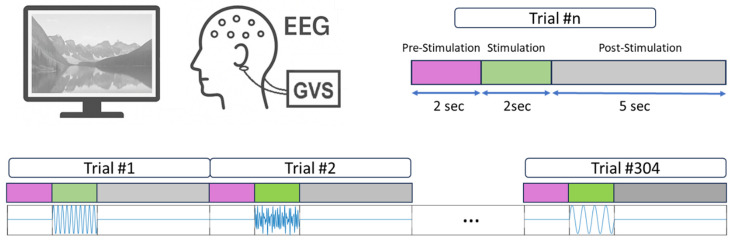
Schematic overview of the experimental paradigm. Each participant underwent a single run consisting of 304 consecutive Galvanic Vestibular Stimulation (GVS) trials, delivered in randomized order without inter-trial pauses. Each trial included three temporal segments: 2 s of pre-stimulation, 2 s of stimulation, and 5 s of post-stimulation. Electroencephalogram (EEG) signals were recorded while participants viewed neutral nature scenes on a monitor. The figure illustrates both the structure of individual trials and the full run timeline (Trial #n means the *n*-th trial).

Figure 1 illustrates the data acquisition setup, including the administration of GVS stimuli and simultaneous EEG recording.

### 2.1. Experimental Design and Data Acquisition

The study included two groups: five healthy control participants and five individuals with PD. Healthy controls were young adults (mean age 23.8 ± 1.6 years, 3 females) with no neurological conditions and no medication use. The PD group consisted of older adults (mean age 71.4 ± 6.0 years, 2 females), with estimated disease duration ranging from approximately 1 to 13 years based on year of diagnosis. All PD participants were tested in their usual ON-medication state (Levodopa/Carbidopa; two participants also taking Entacapone). The two groups were not age-matched, as healthy controls were used solely to define the unstimulated reference distribution in the classifier space rather than for direct EEG group comparisons.

During the experiment, participants passively viewed neutral images sourced from the Geneva Affective Picture Database (GAPED) [20] to remain engaged and prevent boredom. To minimize potential emotional interference with EEG responses to GVS, only neutral images depicting inanimate objects were used. Crucially, image presentation was not synchronized with stimulation events, thereby avoiding unintended temporal associations.

Each participant completed 304 trials, with each trial corresponding to a unique GVS stimulus, including one sham trial without stimulation. The remaining 303 stimuli spanned a diverse set of waveform and frequency characteristics, including direct current (DC), pure sinusoids ranging from 0.5 Hz to 200 Hz, amplitude modulation (AM), chirp signals, chirp-AM, frequency modulation (FM), phase–amplitude coupling (PAC), multisine signals, and binary-modulated patterns. A comprehensive list of stimuli is provided in Appendix A.1.

Each trial lasted nine seconds and followed a consistent structure: a two-second pre-stimulation baseline, a two-second active stimulation interval, and a five-second post-stimulation recovery period (no stimulation). The timing structure of a single trial is depicted in Figure 1, consisting of three segments: 2 s of pre-stimulation, 2 s of stimulation, and 5 s of post-stimulation. Additionally, the figure presents the full run timeline comprising all 304 consecutive trials delivered during the experimental paradigm.

Trials were permuted and then presented sequentially in a single experimental session, with no explicit cues marking transitions between stimuli, ensuring participants remained unaware of stimulus changes. This design enabled the investigation of how distinct stimulation patterns modulate resting-state neural activity, with the broader aim of identifying individualized stimulation protocols tailored to each participant’s neural profile. Event markers indicating stimulus onset were systematically recorded for subsequent analysis.

Prior to the main experiment, participants underwent a GVS thresholding procedure and EEG setup to ensure consistency and data quality. EEG signals were acquired using a 64-channel Quik-Cap Hydro Net (Compumedics Neuroscan, Charlotte, NC, USA) compatible with SynAmps 2/RT and Neuvo amplifiers (Neuroscan, Sterling, VA, USA). The cap included four integrated bipolar leads for vertical and horizontal electrooculography (VEOG, HEOG), as well as electrocardiography (ECG) and electromyography (EMG). Electrode placement adhered to the extended 10–20 system, ensuring standardized coverage across participants. Data were extracted from 36 EEG electrodes spanning frontal, central, parietal, occipital, and temporal regions, along with VEOG and HEOG channels.

All stimulation waveforms were generated in MATLAB R2023b at a sampling frequency of 1000 Hz, and no additional filtering was applied to the synthesized signals. GVS was delivered using a STMISOLA constant current stimulator (BIOPAC^®^ Systems Inc., Goleta, CA, USA), capable of generating arbitrary waveforms. (Note that “constant-current” in this context refers to delivering a given current relatively independent of the current impedance, not that the current delivered was constant). For each participant, Ag/AgCl laminated electrodes (41 mm diameter, with an 11 mm carbon composition contact area) were positioned over the mastoid processes. Electrode placement was verified by applying a 1 Hz sinusoidal stimulus with gradually increasing current amplitudes until the participant reported a gentle rocking sensation.

To ensure subthreshold stimulation, a systematic thresholding protocol was employed. Starting at a baseline current of 0.1 mA, a pink noise stimulus was incrementally increased in 0.05 mA steps over 15 s intervals until participants reported a mild tingling sensation localized to the electrode sites. Once the perceptual threshold was identified, the current was reduced below threshold and then reintroduced to confirm the boundary. All subsequent stimuli were delivered at subthreshold intensity levels.

### 2.2. Preprocessing

To ensure reliable EEG analysis, we employed a multi-stage preprocessing pipeline previously introduced in our earlier work. Raw EEG signals (N = 41, fs = 1000 Hz) were first filtered to remove low-frequency drifts, line noise, and DC offsets, followed by re-referencing with the common average reference (CAR). Non-EEG channels were excluded, and the data were segmented into trials comprising pre- and post-stimulation intervals, resulting in 304 trials across EEG, EOG, and ECG channels.

Artifact removal was performed in two main stages. First, GVS artifacts were suppressed using PCA-based regression, followed by Canonical Correlation Analysis (CCA) to decompose the signals and extract features sensitive to residual GVS and ocular artifacts. Support Vector Machine (SVM) classifiers (implemented in MATLAB R2023a) trained on manually labeled data were then applied to automatically identify and reject artifact-related components, with synthetic oversampling (SMOTE) [21] used to address class imbalance. In the second stage, Independent Component Analysis (ICA) with the CoM_2_ algorithm [22] was performed on the CCA-cleaned signals, and a similar feature-based SVM approach was used to detect electromyographic (EMG) artifacts.

The final cleaned EEG signals were reconstructed after removing contaminated components, followed by residual bandstop and low-pass filtering. Visual inspection of the principal components ensured quality control, yielding artifact-reduced data suitable for subsequent analyses. Full implementation details of this preprocessing workflow are available here: [23].

### 2.3. Proposed Method

#### 2.3.1. Data Segmentation and Feature Extraction

Each trial was divided into three intervals: a 2 s pre-stimulation baseline, a 2 s stimulation window, and a 5 s post stimulation period. EEG biomarkers were extracted from central electrodes (FC5, FC1, FCz, FC2, FC6, C3, Cz, C4, CP5, CP1, CPz, CP2, CP6), which provide reliable coverage of sensorimotor regions commonly implicated in Parkinson’s disease.

A comprehensive set of 16 biomarkers was computed, spanning five physiological domains: phase–amplitude coupling (BG-PAC) [3,11], beta waveform shape (Peak-Trough Asymmetry, Rise-Decay Ratio) [18,19], aperiodic activity (Exponent, Offset) [14,15,24], beta burst properties (Average Burst Duration, Amplitude, Energy) [12,13,25], band power metrics (Beta Power, Relative Beta Power), and signal complexity measures (Petrosian Fractal Dimension, Hjorth Mobility, Hjorth Complexity, Spectral Entropy, Approximate Entropy, Sample Entropy) [16,17,26]. These biomarkers are listed in Table 1.

All biomarkers were included in the analysis to capture complementary aspects of neural activity. To ensure consistency across intervals and avoid bias due to unequal window lengths, all biomarkers were computed using a standardized 2 s analysis window. The 5 s post-stimulation interval was segmented into four overlapping 2 s windows (1 s overlap). Biomarkers were computed for each window and then averaged, yielding post-stimulation estimates directly comparable to those from the PreStim and Stim intervals.

For each trial, biomarker values were averaged across central electrodes to obtain a robust estimate of the subject’s overall neural response. This spatial averaging reduces channel level noise and enhances the stability of biomarker estimates.

#### 2.3.2. Classification Framework

Pre-stimulation intervals served as the reference for classification. A total of 304 pre-stimulation trials from each of the five healthy subjects were labeled as the healthy class, and 304 pre-stimulation trials from each of the five Parkinson’s patients were labeled as the PD class. A leave-one-subject-out cross-validation strategy (10-fold) was employed, where trials from a single subject were used for validation while the remaining trials were used to train a LASSO classifier.

LASSO was selected because it provides a sparse and interpretable linear model suitable for small datasets with correlated biomarkers. Its L1-regularization mitigates multicollinearity by shrinking less informative coefficients, reducing overfitting and improving generalizability. This yields a stable, physiologically interpretable weight vector that defines a single discriminative axis for quantifying stimulation-related shifts.

The LASSO model was trained using the 1SE criterion, and the coefficients from each fold were stored. The average coefficients across 10 folds were used to construct the final trained model, $w_{LASSO}\in R^{N_{b}}$, where $N_{b}=16$ is the number of biomarkers.

The trained model was applied to all pre-stimulation trials, and each trial received a score in the new feature space, denoted as $Score_{preStim}(subj,stim)$, where subj=1,…,10 represents subjects (five healthy and five Parkinson’s patients), and Stim=1,…,304 represents stimulus trials. The reference score for pre-stimulation is computed as the mean across all trials of all healthy subjects as shown in Equation (1):

(1) $$Score_{preStim,HC}=\frac{1}{304\times 5}\sum_{subj=1}^{5}\sum_{stim=1}^{304}Score_{preStim}(subj,stim)$$

#### 2.3.3. Evaluation of Stimulation Trials

The trained model was then applied to stimulation intervals, generating scores $Score_{Stim}(subj,stim)$ for each subject and each trial (i.e., subj=1,…,10 and stim=1,…,304). The central idea is that an effective stimulus should yield a score close to the “pre-stimulation reference”, $Score_{preStim,HC}$. The distance from the reference score for each Parkinson’s subject and trial is computed as Equation (2):

(2) $$Dist_{Stim}\left(subj,stim\right)=\;\left|Score_{Stim}\left(subj,stim\right)-Score_{preStim,HC}\right|$$

Based on this distance measure, stimuli were then ranked for each Parkinson’s subject from best to worst. However, since each stimulus occurs only once per subject, it was beneficial to incorporate data from all five Parkinson’s subjects to improve stimulus ranking.

#### 2.3.4. Ranking of Stimuli Across Subjects

To consolidate information across the five PD subjects and obtain a unified measure of stimulus effectiveness, we introduced a Stimulus Evaluation Score (SES) based on a Multi-Criteria Decision Analysis (MCDA) framework. As described earlier, for each subject and each stimulus, we computed a distance value $Dist_{Stim}\left(subj,stim\right)$ which quantifies how far the stimulation-interval EEG score deviates from the healthy reference point in the classifier space. Smaller values indicate a more desirable shift toward healthy-like EEG patterns.

Because each stimulus was presented only once per subject, individual measurements are inherently noisy. To reduce the influence of subject-specific variability and to integrate information across subjects, we constructed a 304×5 matrix whose entries were the inverse distances, $InvDist\left(subj,stim\right)=1/Dist_{Stim}(subj,stim)$, so that larger values correspond to more desirable responses. For each subject (i.e., each column), values were normalized to ensure comparability across subjects.

This normalized matrix served as the “decision matrix” for MCDA. MCDA provides a principled framework for combining multiple, potentially heterogeneous criteria, in this case, the responses of five PD subjects, into a single interpretable score. Unlike single-objective ranking methods, MCDA identifies an ideal solution (the maximum normalized value across subjects) and an anti-ideal solution (the minimum normalized value), and evaluates each stimulus based on its Euclidean distance to these reference points. The relative closeness to the ideal solution was then defined as the Stimulus Evaluation Score, $SES\left(stim\right),\;stim=1,\ldots ,304$.

Stimuli were finally ranked in descending order of SES. This approach provides a transparent and reproducible method for aggregating multi-subject EEG responses and identifying waveform classes that consistently shift PD EEG patterns toward healthy-like activity.

#### 2.3.5. Grouping and Statistical Analysis

Because each stimulus was presented only once per subject, direct ranking of individual stimuli is statistically underpowered. To enable more reliable comparisons, the 304 stimuli were grouped into 37 categories based on waveform morphology and frequency content (Appendix A.2).

For each PD subject, we computed the distance metric $Dist_{Stim}\left(subj,stim\right)$ (Equation (1)), which quantifies the deviation of each stimulation-interval EEG response from the healthy reference score. Stimuli were then ranked within each subject according to this distance, yielding an ordinal ranking $Rank_{subj}(stim)$.

To evaluate performance at the group level, we calculated the median rank of all stimuli within each group for each subject, denoted as $MedianRank_{subj}(Stim\;Group)$. This aggregation reduces the influence of single-trial noise and provides a more stable estimate of group-level effectiveness.

Inter-subject agreement regarding the relative ordering of stimulus groups was assessed using Kendall’s coefficient of concordance (W), a non-parametric measure of consistency across raters. In addition, a one-tailed Wilcoxon rank sum test was used to compare each group’s ranks against the pooled ranks of all other groups, identifying waveform categories with significantly lower (i.e., more favourable) median ranks. Multiple comparisons were controlled using the Benjamini–Hochberg procedure (implemented in MATLAB R2023a).

Together, these analyses provide a principled framework for identifying stimulus categories that consistently shift EEG biomarkers toward healthy-like patterns across subjects.

#### 2.3.6. Validation Using Post-Stimulation Trials

To assess whether the stimulation-interval findings reflected genuine stimulation-related effects rather than analysis artifacts or general signal variability, the entire analysis pipeline was repeated using post-stimulation data. Because the neural effects of subthreshold GVS are expected to dissipate rapidly after stimulus offset, we hypothesized that post-stimulation results, such as the ranking of stimulus groups or the degree of agreement across subjects, would not exhibit the structured patterns observed during stimulation. This comparison served as a control analysis to verify that the effects identified during the stimulation interval were specifically time-locked to the stimulus.

## 3. Results

**Figure 2:**
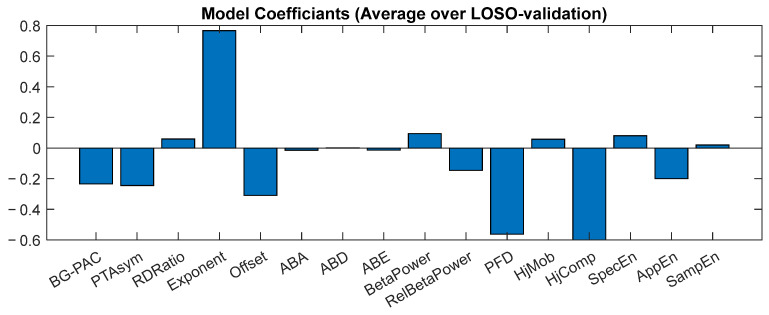
Average coefficients of the LASSO model trained to distinguish healthy and Parkinsonian trials using pre-stimulus EEG data. Coefficients were computed using Leave-One-Subject-Out cross-validation and reflect the multivariate discriminative contribution of the biomarker set.

The LASSO classifier was first trained on pre-stimulation EEG data to distinguish healthy from Parkinsonian trials. The resulting coefficients, averaged across folds of the leave-one-subject-out cross-validation procedure, are shown in Figure 2. Because several biomarkers exhibit inter-correlations, individual LASSO coefficients should not be interpreted in isolation. Instead, the classifier captures a multivariate combination of features that jointly contribute to discrimination. Supplementary Material provides additional analyses illustrating the distribution of individual biomarkers and their relationships.

Classification performance across the three EEG intervals, PreStim, Stim, and PostStim, is summarized in Table 2. Accuracy values ranged from approximately 80% to 90%. As expected, the highest accuracy was obtained for the PreStim trials (Acc =90.33%), which were used for model training. Accuracy decreased during the Stim interval (Acc =84.05%), consistent with stimulation-related deviations from baseline activity, and increased again during the PostStim interval (Acc =88.68%). These results indicate that post-stimulation activity more closely resembles baseline, whereas stimulation introduces transient changes that differentiate the Stim interval from both pre- and post-stimulation periods.

**Figure 3:**
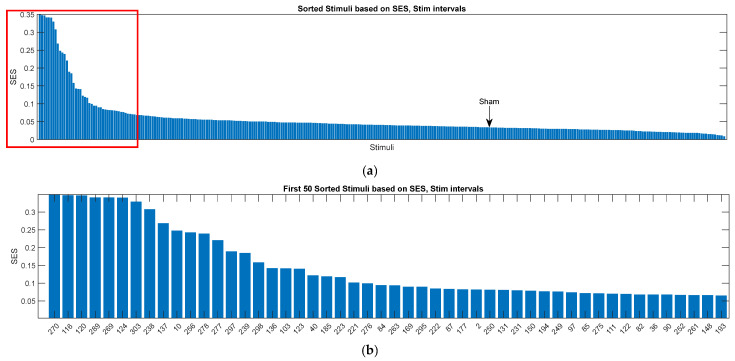
Stimuli ranked by Stimulus Evaluation Score (SES) values computed during the Stim interval. (**a**) SES values shown without indices for clarity; the red square highlights the first 50 stimuli, which are shown in zoomed form in subfigure (b). (**b**) Top 50 stimuli with their corresponding indices. See Appendix A.1 for stimulus descriptions.

Stimuli ranked according to their SES values during the Stim interval are shown in Figure 3. Panel (a) illustrates the distribution of SES values, and panel (b) displays the top 50 ranked stimuli along with their indices. The sham condition ranked 210th among the 304 stimuli, placing it near the midpoint of the distribution, consistent with its expected neutral effect.

To assess whether SES values reflected stimulation-locked effects rather than general variability, we examined the relationship between SES values computed during the Stim and PostStim intervals. The Spearman correlation between Stim−SES and PostStim−SES was 0.099 (p=0.085), indicating a weak and statistically non-significant association. This lack of correspondence suggests that SES values derived from the stimulation window capture transient, time-locked neural responses that do not persist into the post-stimulation period.

To further assess the reliability of the findings, we examined the agreement among subjects in their ranking of stimulation categories. This analysis was performed separately for the Stim and PostStim intervals. For each subject and each stimulation category (Appendix A.2), we computed the median rank of the stimuli within that category, yielding one median rank per category per subject. Categories containing only a single stimulus were excluded, resulting in 35 categories included in the analysis.

In parallel, a group level ranking was obtained using the SES metric computed from the combined data across subjects. Stimuli were sorted according to their SES values, and median SES based ranks were calculated for each category. These median values provided a reference ordering of stimulation categories at the group level.

**Figure 4:**
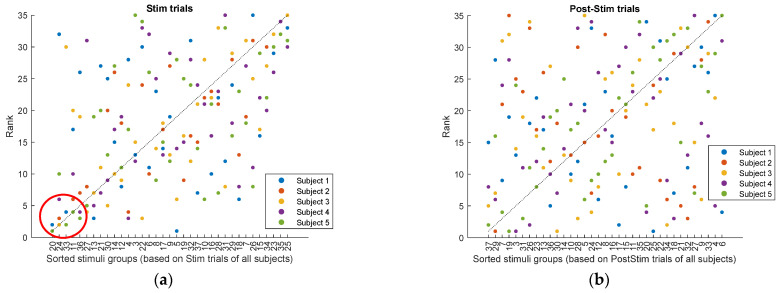
Agreement analysis of stimulation category rankings across subjects. (**a**) Rankings derived from Stim interval data. Categories are sorted along the x axis based on group level SES ranking. Each point represents the rank assigned by one subject; colors denote subjects. Proximity to the diagonal (y=x) indicates stronger agreement, particularly evident at the highest ranked categories (circled) (**b**) Same analysis for PostStim intervals, showing reduced agreement across subjects.

Figure 4 summarizes the agreement analysis. In Figure 4a (Stim interval), the x axis shows stimulation categories sorted by their group level SES ranking, while the y axis shows the rank assigned by each subject. Points lying near the diagonal (y=x) indicate stronger agreement. Although variability is present, clearer alignment is observed for the highest-ranked categories. In contrast, Figure 4b (PostStim interval) shows greater dispersion, indicating weaker agreement across subjects once stimulation has ceased.

Inter-subject consistency was quantified using Kendall’s coefficient of concordance (W). During the Stim interval, Kendall’s W=0.46 (p=2×10−5), indicating moderate and statistically significant agreement among subjects. During the PostStim interval, Kendall’s W=0.18 (p=0.624), reflecting low and non-significant agreement. These results suggest that consistent subject-level ranking patterns emerge only during active stimulation (Table 3).

**Figure 5:**
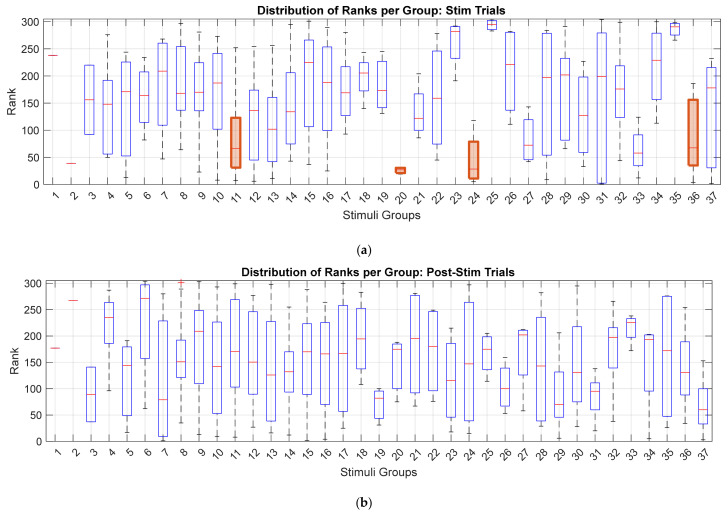
Boxplots of SES values across stimulation categories. (**a**) SES distributions during the Stim interval. Statistical significance was assessed using a one-tailed Wilcoxon rank-sum test with Benjamini–Hochberg FDR correction (*q* < 0.1). Four categories were identified as significantly effective; the orange boxes correspond to these effective stimulation categories that exhibited significantly lower ranks. (**b**) SES distributions during the PostStim interval, where no categories reached significance. The red ‘+’ symbols indicate outlier data points.

Figure 5 presents the final identification of effective stimulation categories. For the Stim interval (Figure 5a), SES values were compared across categories using a one-tailed Wilcoxon rank-sum test to determine whether a category’s ranks were significantly lower than the distribution of all ranks. Multiple comparisons were corrected using the Benjamini–Hochberg false discovery rate procedure (q<0.1). Four stimulation categories were identified as statistically significant:

- Group 11 (Sinusoidal beta 2),
- Group 20 (Multisine MVS) [9],
- Group 33 (FM with 75 Hz carrier),
- Group 36 (Phase–Amplitude Coupling).

Figure 5b shows the corresponding analysis for the PostStim interval, where no stimulation category reached statistical significance.

To further characterize stimulation-related effects, two EEG biomarkers, Peak-Trough Asymmetry and Relative Beta Power, were analyzed across the three temporal intervals (PreStim, Stim, and PostStim). Biomarkers were computed for all trials belonging to the selected stimulation groups, separately for five PD participants and five HCs, across 36 EEG channels.

For each biomarker, paired comparisons between temporal intervals (PreStim vs. Stim, Stim vs. PostStim, and PreStim vs. PostStim) were performed using paired *t*-tests. Multiple-comparison correction was applied using the Benjamini–Hochberg FDR. Analyses were conducted independently for PD and HC groups.

**Figure 6:**
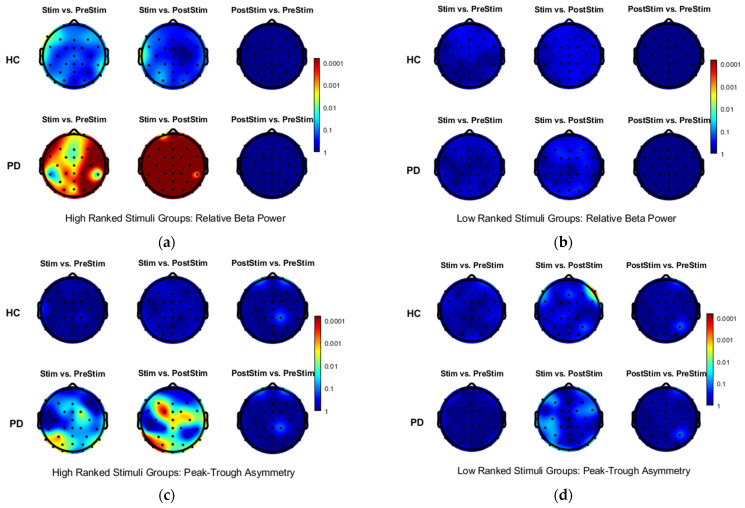
Panels (**a**,**b**) show −log_10_(*q*-value) maps for Relative Beta Power; panels (**c**,**d**) show −log_10_(*q*-value) maps for Peak–Trough Asymmetry. Each column compares Stim vs. PreStim, Stim vs. PostStim, and PostStim vs. PreStim intervals across 36 EEG channels. Rows correspond to healthy controls (HC, **top**) and Parkinson’s patients (PD, **bottom**). Significant effects were observed only in PD participants and only for high-ranked stimulation groups: Relative Beta Power changes were widespread across the scalp, whereas Peak–Trough Asymmetry effects were localized to central and occipital channels. Low-ranked groups and all HC comparisons showed no significant differences. Warmer colors indicate stronger significance (lower *q*-values). Dots indicate EEG electrode locations.

Figure 6a shows the −log_10_(*q*-value) maps for Relative Beta Power in HC (top row) and PD participants (bottom row) for the three interval comparisons. Figure 6c presents the corresponding results for Peak-Trough Asymmetry. The same analysis was repeated for trials from low-ranked stimulation groups, with results shown in Figure 6b,d.

As illustrated in Figure 6, statistically significant interval-dependent changes were observed almost exclusively in PD participants and only for trials from high-ranked stimulation groups. These effects were most prominent in the PreStim vs. Stim and Stim vs. PostStim comparisons. Relative Beta Power exhibited widespread significance across the scalp, whereas Peak-Trough Asymmetry showed more localized effects, primarily over central and occipital regions.

No significant differences were detected between PreStim and PostStim intervals under any condition, and no statistically significant effects were observed in healthy controls for either biomarker, any stimulation group, or any interval pairing.

## 4. Discussion

This study introduces an EEG based framework for evaluating the immediate neural effects of GVS in PD. The approach is motivated by a practical challenge in neuromodulation research: the difficulty of behaviourally testing large numbers of stimulation waveforms in clinical populations, who may tolerate testing for only relatively brief periods. By using resting-state EEG biomarkers as a multidimensional proxy for cortical state, the framework enables rapid, scalable screening of candidate stimulation patterns prior to behavioural validation.

The composite score used in this work integrates multiple EEG biomarkers spanning oscillatory, aperiodic, burst-related, and complexity-based features. Because several biomarkers are correlated, the LASSO-derived score should be interpreted as a multivariate representation rather than as evidence for the importance of any individual feature. Supplementary analyses further suggested that no single biomarker consistently separates PD from healthy controls, reinforcing the value of a combined metric for capturing broader cortical dynamics (see Supplementary Materials).

### 4.1. Interpretation of Effective Stimulation Categories

The analysis identified several stimulation categories that produced larger shifts in the composite EEG score during the stimulation interval. These included beta-frequency sinusoids, multisine signals, frequency-modulated signals, and PAC-modulated waveforms. These findings indicate that certain waveform families exerted more pronounced effects on the multivariate EEG metric during the resting-state conditions tested.

It is important to emphasize that the present results do not necessarily establish mechanistic relationships between waveform properties and specific neural processes. The composite score reflects a combination of correlated biomarkers, and the observed ranking of stimulation categories should be interpreted as an empirical outcome of this multivariate metric rather than as evidence for underlying physiological mechanisms. Because each waveform was presented only once per subject, the grouping strategy was essential for identifying consistent patterns across participants. The categories highlighted here therefore represent promising candidates for further investigation rather than definitive optimal waveforms.

### 4.2. Temporal Dynamics of Stimulation Effects

One practical advantage of electrical stimulation, compared with other non-invasive neuromodulation approaches such as transcranial magnetic stimulation (TMS) or transcranial ultrasound (TUS), is that it can be delivered reliably using portable hardware and does not depend on substantial post-stimulation after-effects. In the present study, stimulation-related changes in the composite EEG score were confined to the active stimulation period. No significant differences were observed between pre- and post-stimulation intervals, and inter-subject agreement in ranking stimulation categories declined markedly once stimulation ceased. This pattern indicates that the major neural effects of subthreshold GVS were transient and state-dependent, emerging only during stimulus delivery. These findings are consistent with prior reports showing that the behavioural and neural effects of vestibular stimulation typically occur during stimulation and diminish rapidly thereafter, with limited evidence for sustained after-effects at low intensities (e.g., [27,28]). Accordingly, the absence of post-stimulation carryover in our data supports the interpretation that the observed EEG changes reflect immediate, stimulation-locked modulation rather than nonspecific fluctuations in resting-state activity.

From a translational perspective, this temporal specificity suggests that continuous or periodically delivered stimulation may be necessary to achieve sustained functional benefits. It also reinforces the value of analyzing stimulation-interval data as the primary window in which GVS exerts measurable cortical effects under resting-state conditions.

### 4.3. Context Dependence of EEG Biomarkers

The behavior of individual EEG biomarkers is known to depend on the experimental context. Features such as beta power and phase–amplitude coupling often show greater modulation during movement or task engagement [29,30], whereas aperiodic and entropy-based measures may more accurately reflect resting state dynamics [15,17,24,26]. Because all data in the present study were collected at rest, the relative contributions of biomarkers to the composite score reflect their resting state properties and may not generalize directly to active or task-based conditions. Future work incorporating both rest and movement paradigms will be necessary to determine the stability and context dependence of these biomarkers.

### 4.4. Methodological Strengths and Limitations

This study has several methodological strengths that support the validity of the findings despite the small cohort size. First, the framework is designed to be scalable and adaptable: the stimulus-selection pipeline can be applied on a per-participant basis, meaning that increasing the sample size would extend rather than alter the proposed pathway. Second, although each waveform was sampled sparsely (with only a single trial per subject) the grouping strategy, which aggregated 304 unique stimuli into 37 waveform categories, enabled robust statistical comparisons and revealed consistent group-level patterns. This approach mitigated the limitations of single-trial sampling and provided a principled way to extract reliable information from a large stimulus space.

Another strength is that all stimulation was delivered at subthreshold intensities. Participants did not consciously perceive the stimulation, controlling for the placebo effect, yet measurable EEG changes were observed. This demonstrates that low-intensity vestibular input is sufficient to modulate cortical activity, an encouraging property for long-term tolerability and future clinical translation.

At the same time, several limitations must be acknowledged. The small sample size limits generalizability, and replication with larger cohorts will be essential. Each waveform was tested only once per subject, constraining the reliability of single trial estimates; repeated presentations will be necessary to confirm waveform-specific effects. The study did not include behavioural outcomes, so the relationship between EEG-based changes and functional improvements remains inferential.

All data were collected under resting-state conditions, meaning that biomarker behavior, and therefore the composite score, may differ during movement or task engagement. Features such as beta power and phase–amplitude coupling often show stronger modulation during active states, whereas entropy and aperiodic metrics may better reflect resting dynamics. As such, the biomarker rankings observed here reflect their utility in rest and may not generalize to task-based paradigms, and ultimately observable behavioural changes. Future studies incorporating both rest and movement conditions will be important for assessing the stability, specificity, and complementarity of biomarkers across behavioural contexts.

### 4.5. Translational Outlook

Despite its limitations, the proposed framework offers a practical pathway for accelerating the identification of candidate GVS waveforms. By combining systematic stimulus design with EEG based evaluation, the approach reduces reliance on labor-intensive behavioural testing and provides a principled method for narrowing the search space. A potential translational trajectory involves (1) EEG-based screening to identify promising waveform categories, (2) behavioural validation to confirm functional relevance, (3) integration into adaptive or closed-loop systems that tailor stimulation to individual neural signatures and (4) ultimate assessment of efficacy in clinical trials.

This progression aligns with precision medicine goals and highlights the potential for biologically informed optimization of GVS protocols in PD. The present study provides an initial foundation for such efforts, offering a scalable and interpretable framework for early-stage screening of neuromodulatory stimuli.

## 5. Conclusions

This study presents a scalable EEG-based framework for evaluating the immediate neural effects of a large library of GVS waveforms in Parkinson’s disease. By screening more than 300 unique stimuli and quantifying their impact using a multivariate composite EEG metric, we identified several stimulation categories that produced more pronounced changes during the stimulation interval under resting-state conditions.

Although behavioural outcomes were not assessed, the findings represent an early step toward developing an efficient, non-invasive screening approach that can substantially reduce the burden of behavioural testing in future neuromodulation studies. The framework is designed to be adaptable and extensible, providing a structured pathway for narrowing the stimulus space before behavioural validation and for future integration into individualized stimulation strategies.

Overall, this work establishes a methodological foundation for high-throughput evaluation of GVS waveforms and offers a practical starting point for subsequent studies aimed at linking EEG-based screening to functional outcomes and advancing precision-oriented neuromodulation in Parkinson’s disease.

## Figures and Tables

**Table 1:** List of Electroencephalogram (EEG) biomarkers used for stimulus evaluation, grouped by physiological domain.

Biomarker Group	Biomarker	Abbreviation
Phase–Amplitude Coupling	Beta–Gamma Phase–Amplitude Coupling	BG-PAC
Beta waveform	Peak-Trough Asymmetry	PTAsym
	Rise-Decay Ratio	RDRatio
Aperiodic	Aperiodic Exponent	Exponent
	Aperiodic Offset	Offset
Beta Burst	Average Burst Duration	ABD
	Average Burst Amplitude	ABA
	Average Burst Energy	ABE
Band Power	Beta Power	BetaPower
	Relative Beta Power	RelBetaPower
Signal Complexity	Petrosian Fractal Dimension	PFD
	Hjorth Mobility	HjMob
	Hjorth Complexity	HjComp
	Spectral Entropy	SpecEn
	Approximate Entropy	AppEn
	Sample Entropy	SampEn

**Table 2:** Classification accuracy of the LASSO model across the three EEG intervals (PreStim, Stim, and PostStim).

Trials	PreStim	Stim	PostStim
Accuracy (%)	90.33	84.05	88.68

**Table 3:** Inter subject agreement in stimulation category rankings during Stim and PostStim intervals.

	Stim Intervals		PostStim Intervals	
Kendall’s W value	0.46	Moderate level of agreement	0.18	low agreement
*p*-value	$2\times 10^{-5}$	Statistically significant	0.624	not statistically significant

## Data Availability

De-identified data will be made available via written request to the corresponding author.

## Supplementary Materials

The following supporting information can be downloaded at: https://www.mdpi.com/article/10.3390/biomedicines14020352/s1. Figure S1: Biomarker distributions across PreStim, Stim, and PostStim intervals for high ranked stimuli (Groups 11, 20, 33, 36). (a) PD, (b) HC. Violin plots show that several biomarkers in PD subjects differ significantly during the Stim interval compared to both PreStim and PostStim, while PreStim and PostStim do not differ. No significant changes appear in healthy controls. Figure S2: Within trial deviations from baseline across Stim and PostStim windows for high ranked stimuli. Each curve shows biomarker changes relative to PreStim. Thin lines show the average of trials for each subject (blue: PD and red: HC). Thick lines show the average of HC subjects and PD subjects. Figure S3: Probability density functions (PDFs) of individual biomarkers for PreStim trials across subjects. Thin blue and red curves show subject level PDFs for PD and healthy participants, respectively; thick curves show group level PDFs. Figure S4: (a) PDFs of the composite biomarker obtained by projecting all features onto the optimized LASSO weight vector. (b) Variance of the composite biomarker, overall vs. within-group vs. within-subject.

## Abbreviations

The following abbreviations are used in this manuscript:

PD	Parkinson’s Disease
GVS	Galvanic Vestibular Stimulation
MCDA	Multi-Criteria Decision Analysis
DBS	Deep Brain Stimulation
PAC	Phase–Amplitude Coupling
EEG	Electroencephalogram
DC	Direct Current
AM	Amplitude Modulation
FM	Frequency Modulation
LASSO	Least Absolute Shrinkage and Selection Operator
SVM	Support Vector Machine
SMOTE	Synthetic Minority Oversampling Technique

## Appendix A

### Appendix A.1

**Table A1:** List of all stimuli.

Category	Stimuli Description	Stimulus IDs
Baseline & DC	Sham, Pink Noise, DC + 1, DC − 1	1–4
Chirp Signals	Delta, Theta, Alpha, Beta, Gamma, High 1–3 (50–200 Hz)	5–12
Sinusoidal (AC)	0.5 Hz to 200 Hz in ~1 Hz increments	13–213
Multisine	Theta, Alpha, Beta, Gamma, High 1–3 (50–200 Hz), MVS variants	214–223
AM	Carrier: 75/100/125 Hz; Envelope: 9/11/16/25 Hz	224–247
Chirp AM	Carrier: 75/100/125 Hz; Chirp: Delta-Gamma	248–275
FM Signals	Carrier: 75/100/125 Hz; Modulation: 9/11/16/25 Hz	276–287
PAC Signals	Phase-Amplitude Coupling: combinations of Delta-Gamma bands	288–297
BN-Modulated Signals	Band-limited noise modulated: Delta, Theta, Alpha, Beta, Gamma, High 1–2	298–304

### Appendix A.2

**Table A2:** List of stimuli groups.

Group	Group Name	Stimulus Types	Stimulus Numbers
1	Sham	Sham	1
2	Pink noise	Pink noise	2
3	DC	DC + 1, DC − 1	3, 4
4	Chirp_Low	Chirp Delta, Theta, Alpha, Beta	5–9
5	Chirp_High	Chirp High 1, 2, 3 (50–200 Hz)	10–12
6	Sinusoidal up to 1.5 Hz	Sinusoidal 0.50–1.40 Hz	13–16
7	Sinusoidal Delta	Sinusoidal 1.70–4.00 Hz	17–25
8	Sinusoidal Theta	Sinusoidal 4.30–7.90 Hz	26–38
9	Sinusoidal Alpha	Sinusoidal 8.20–12.90 Hz	39–55
10	Sinusoidal Beta 1	Sinusoidal 13.20–20.00 Hz	56–79
11	Sinusoidal Beta 2	Sinusoidal 20.30–30.00 Hz	80–113
12	Sinusoidal Gamma 1	Sinusoidal 31.70–50.40 Hz	114–125
13	Sinusoidal Gamma 2	Sinusoidal 52.10–70.80 Hz	126–137
14	Sinusoidal Gamma 3	Sinusoidal 72.50–99.70 Hz	138–154
15	Sinusoidal High 1	Sinusoidal 101.40–135.40 Hz	155–175
16	Sinusoidal High 2	Sinusoidal 137.10–169.40 Hz	176–195
17	Sinusoidal High 3	Sinusoidal 171.10–200.00 Hz	196–213
18	Multisine (Low)	Multisine Theta, Alpha, Beta, Gamma	214–217
19	Multisine (High)	Multisine High 1, 2, 3 (50–200 Hz)	218–220
20	Multisine MVS	MVS-4, MVS-L, MVS-S	221–223
21–23	AM (Imp. 1) C: 75/100/125 Hz	AM C with E: 9–25 Hz	224–235
24–26	AM (Imp. 2) C: 75/100/125 Hz	AM C with E: 9–25 Hz	236–247
27–29	Chirp AM (Imp. 1) C: 75/100/125 Hz	Chirp: Delta–Gamma Hz	248–261
30–32	Chirp AM (Imp. 2) C: 75/100/125 Hz	Chirp: Delta–Gamma Hz	262–275
33–35	FM C: 75/100/125 Hz	Modulation: 9–25 Hz	276–287
36	PAC	PAC delta-theta to beta–gamma	288–297
37	BN-modulated	BN-modulated Delta to High 2	298–304
