# Supplementary material for: Rapid Profiling of EEG Responses to Non-Invasive Brain Stimulation in Parkinson’s Disease: A Biomarker-Driven Screening Framework
Source: Biomedicines. 2026 Feb 3;14(2):352. doi: 10.3390/biomedicines14020352 (PMC12937774; doi:10.3390/biomedicines14020352)
Supplement: Supplementary file 1 [file biomedicines-14-00352-s001.zip › biomedicines-4064576-supplementary.pdf]

Supplementary Material

SM1. Additional Analysis of PreStim, Stim, and PostStim Intervals

Biomarker Distributions Across Trial Segments (Figure S1)

For the highest-ranked stimulus groups (Groups 11, 20, 33, 36), all 16 EEG biomarkers were evaluated using a linear mixed-effects model with subject and subject×stimulus random effects. FDR-corrected contrasts revealed:

- Significant deviations during the Stim interval relative to both PreStim and PostStim for several biomarkers in PD subjects, including Peak-Trough Asymmetry, Rise-Decay, Beta Power, Relative Beta Power, and Petrosian Fractal Dimension.
- No significant differences between PreStim and PostStim, indicating a rapid return to baseline.
- No systematic changes across intervals in healthy controls.

These results confirm that the observed effects are time-locked to stimulation and not attributable to slow drifts or residual artefacts.

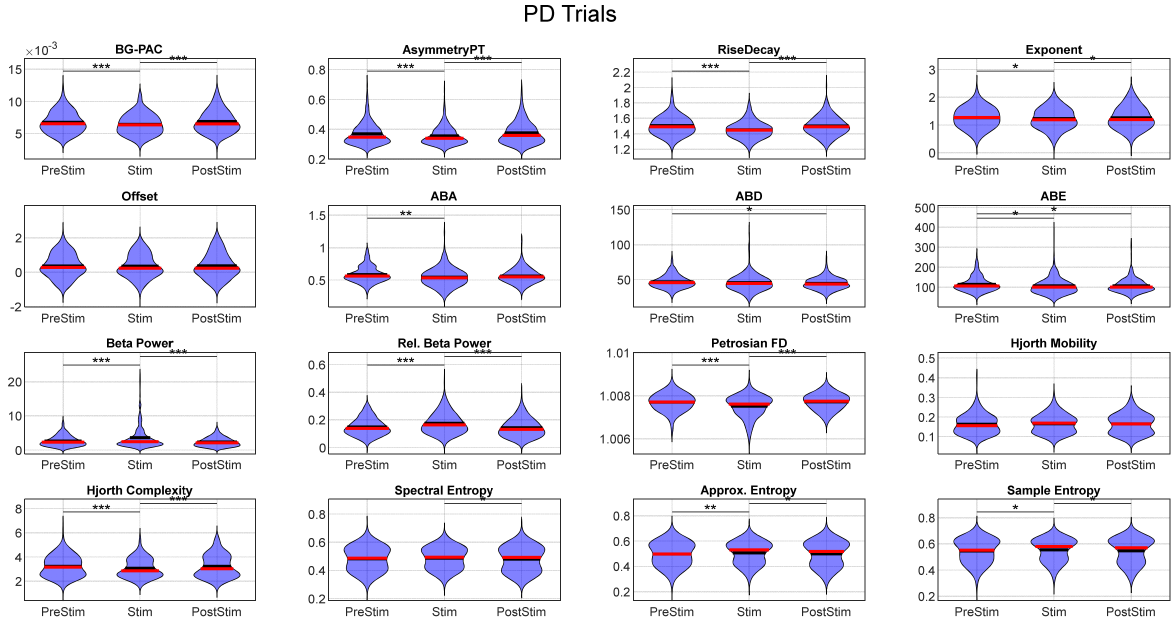

(a)

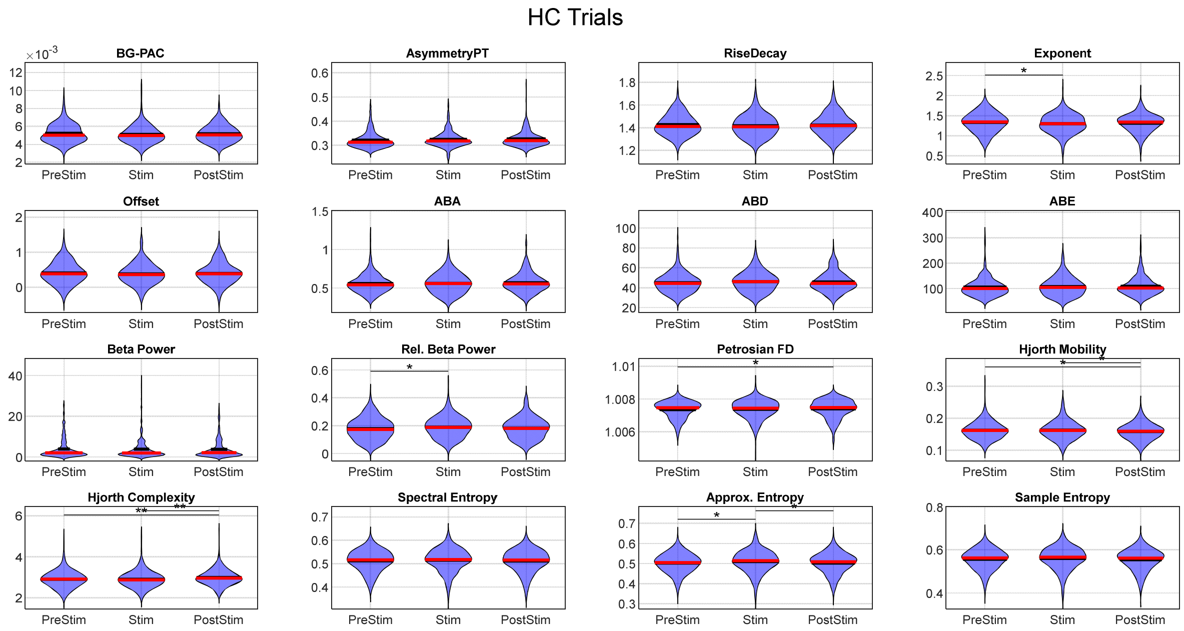

(b)

Figure S1. Biomarker distributions across PreStim, Stim, and PostStim intervals for high-ranked stimuli (Groups 11, 20, 33, 36). (a) PD, (b) HC. Violin plots show that several biomarkers in PD subjects differ significantly during the Stim interval compared to both PreStim and PostStim, while PreStim and PostStim do not differ. No significant changes appear in healthy controls.

SM2. Within-Trial Temporal Trajectories (Figure S2)

To further characterize the temporal profile of stimulation-related changes, deviations from each subject's PreStim baseline were computed across the Stim interval and four consecutive 2-second PostStim windows.

Key Findings

- PD subjects exhibited clear transient deviations during stimulation, often forming a distinct peak or trough depending on the biomarker.
- These deviations rapidly diminished after stimulation offset, returning toward baseline.
- Healthy subjects showed no consistent modulation across any interval.

These findings support the interpretation that the observed effects reflect genuine, short-latency neural responses rather than artefactual contamination.

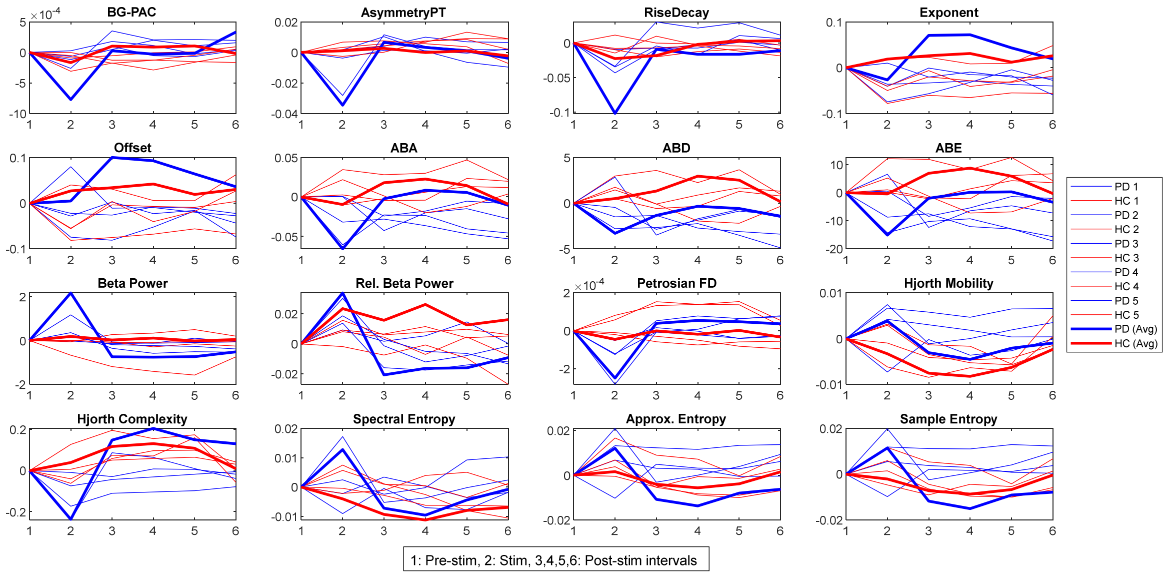

Figure S2. *Within-trial deviations from baseline across Stim and PostStim windows for high-ranked stimuli.* Each curve shows biomarker changes relative to PreStim. Thin lines show the average of trials for each subject (blue: PD and red: HC). Thick lines show the average of HC subjects and PD subjects.

SM3. Distributional Analysis of All 16 EEG Biomarkers (Figure S3)

To evaluate biomarker selection and potential multicollinearity, probability density functions (PDFs) of all 16 biomarkers during the PreStim interval were plotted for each subject and group.

Key Observations

- Individual biomarkers do not consistently separate PD and HC groups.
- Within-group and within-subject variability can be comparable to between-group variability.
- No single biomarker reliably distinguishes the two groups.

This pattern aligns with prior EEG literature, particularly in small cohorts, and underscores the need for a multivariate composite biomarker.

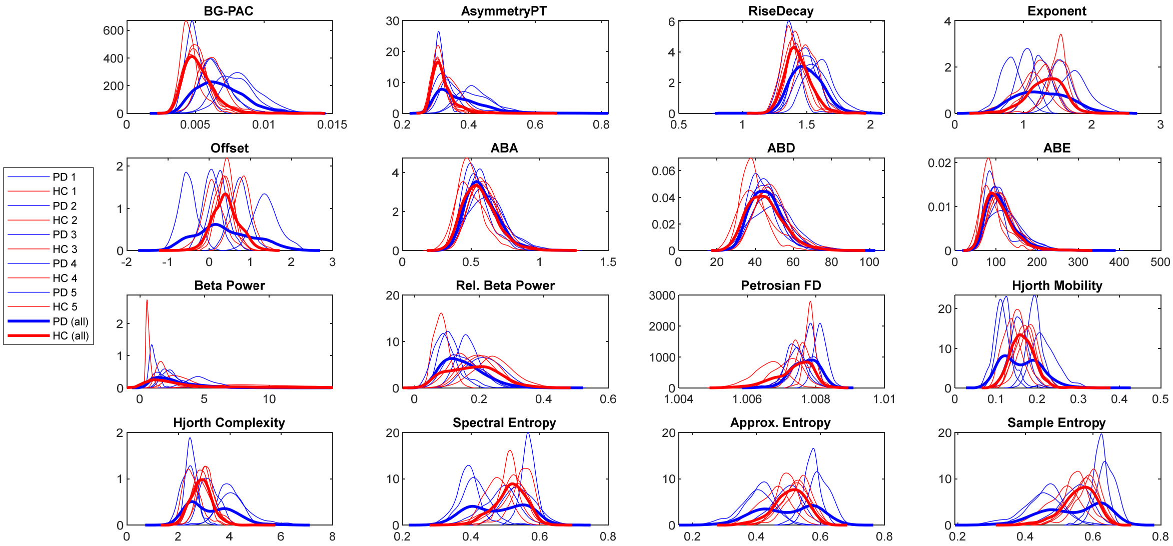

Figure S3. *Probability density functions (PDFs) of individual biomarkers for PreStim trials across subjects.*
Thin blue and red curves show subject-level PDFs for PD and healthy participants, respectively; thick curves show group-level PDFs.

SM4. Composite Biomarker Projection and Variance Decomposition (Figure S4)

To assess whether a multivariate approach better captures group differences, all trials were projected onto the optimized LASSO weight vector.

S4.1. Composite Biomarker PDFs (Figure S4a)

- The composite biomarker shows clear separation between PD and HC at both the subject and group levels.
- This separation is substantially stronger than that observed for any individual biomarker.

S4.2. Variance Decomposition (Figure S4b)

Variance components were quantified, revealing that:

- Between-group variance is substantially larger than within-group and within-subject variance.
- The composite biomarker captures a consistent, directionally aligned difference between PD and HC.

These results justify the use of a data-driven multivariate framework.

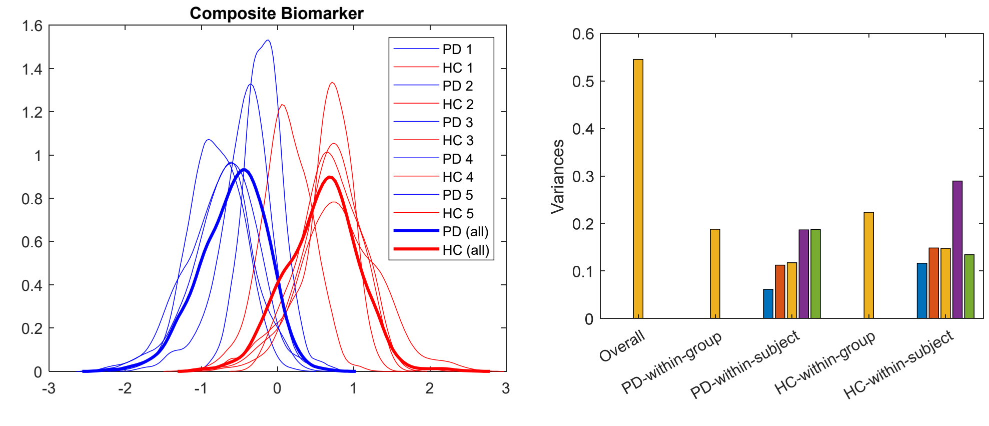

(a) (b)

Figure S4. (a) PDFs of the composite biomarker obtained by projecting all features onto the optimized LASSO weight vector. (b) Variance of the composite biomarker, overall vs. within-group vs. within-subject.
